# Development of core competencies for field veterinary epidemiology training programs

**DOI:** 10.3389/fvets.2023.1143375

**Published:** 2023-04-06

**Authors:** Julio Pinto, Ravi Bandara Dissanayake, Navneet Dhand, Cristina Rojo-Gimeno, Laura Cristina Falzon, Holy Akwar, Rianatou Bada Alambeji, Daniel Beltran-Alcrudo, David Mario Castellan, Karoon Chanachai, Javier Guitian, Angela Hilmers, Fairouz Larfaoui, Leo Loth, Paolo Motta, Harentsoaniaina Rasamoelina, Stephanie Salyer, Sean Shadomy, Cécile Squarzoni, Innocent Rwego, Carmen Varela Santos, Kachen Wongsathapornchai, Caryl Lockhart, Sam Okuthe, Yaghouba Kane, Jeffrey Gilbert, Baba Soumare, Madhur Dhingra, Keith Sumption, Thanawat Tiensin

**Affiliations:** ^1^Food and Agriculture Organization of the United Nations, Rome, Italy; ^2^Food and Agriculture Organization of the United Nations, Liaison Office for the United Nations, Geneva, Switzerland; ^3^Sydney School of Veterinary Science, The University of Sydney, Camden, NSW, Australia; ^4^Ecole Inter-Etats des Sciences et Medecine Vétérinaires, Dakar, Senegal; ^5^Food and Agriculture Organization Regional Office for Europe and Central Asia, Budapest, Hungary; ^6^Institute for Infectious Animal Diseases, Texas A&M University, College Station, TX, United States; ^7^Department of Disease Control/Department of Livestock Development, Ministry of Agriculture and Cooperatives, Bangkok, Thailand; ^8^The Royal Veterinary College, Hatfield, United Kingdom; ^9^Training Programs in Epidemiology and Public Health Interventions Network, Atlanta, GA, United States; ^10^Food and Agriculture Organization Emergency Center for Transboundary Animal Diseases, Hanoi, Vietnam; ^11^Epidemic Surveillance and Alert Management, Indian Ocean Commission, One Health Network, Ebène, Mauritius; ^12^Centers for Disease Control and Prevention, Atlanta, GA, United States; ^13^Centre de Coopération Internationale en Recherche Agronomique pour le Développement, La Réunion, France; ^14^University of Minnesota/One Health Central and Eastern Africa, Makerere University, Kampala, Uganda; ^15^Public Health Capacity and Communication Unit, European Centre for Disease Prevention and Control (ECDC), Stockholm, Sweden; ^16^Food and Agriculture Organization, Emergency Center for Transboundary Animal Diseases, Bangkok, Thailand; ^17^Food and Agriculture Organization of the United Nations, Nairobi, Kenya; ^18^Food and Agriculture Organization of the United Nations Regional Office for Africa, Accra, Ghana

**Keywords:** field veterinary epidemiology, core competencies, workforce capacity development, epidemiological surveillance, field investigation, One Health

## Abstract

A workforce with the adequate field epidemiology knowledge, skills and abilities is the foundation of a strong and effective animal health system. Field epidemiology training is conducted in several countries to meet the increased global demand for such a workforce. However, core competencies for field veterinary epidemiology have not been identified and agreed upon globally, leading to the development of different training curricula. Having a set of agreed core competencies can harmonize field veterinary epidemiology training. The Food and Agriculture Organization of the United Nations (FAO) initiated a collective, iterative, and participative process to achieve this and organized two expert consultative workshops in 2018 to develop core competencies for field veterinary epidemiology at the frontline and intermediate levels. Based on these expert discussions, 13 competencies were identified for the frontline and intermediate levels. These competencies were organized into three domains: epidemiological surveillance and studies; field investigation, preparedness and response; and One Health, communication, ethics and professionalism. These competencies can be used to facilitate the development of field epidemiology training curricula for veterinarians, adapted to country training needs, or customized for training other close disciplines. The competencies can also be useful for mentors and employers to monitor and evaluate the progress of their mentees, or to guide the selection process during the recruitment of new staff.

## Introduction

Field epidemiologists are at the forefront of detecting and responding to disease outbreaks and other public health emergencies, and providing evidence-based recommendations for decision-makers (1–3). To address the increasing demand for such skills, several countries have established field epidemiology training programs (FETPs) for Public Health professionals with the support of the United States Center for Disease Control and Prevention (US CDC) (4–6). Traditionally, FETPs were 2-year advanced programs hosted within the Ministries of Health (6), although they now generally follow a three-tier model—frontline, intermediate and advanced levels—to allow for capacity-building at three hierarchical levels (7). The frontline level is a 3-month long training program aiming to prepare public health workers to work at local and community levels. The intermediate tier training lasts nine months and prepares public health workers to provide support at the district level. The advanced tier is an intensive two-year training for experienced epidemiology health professionals and prepares them for leadership roles within the Ministries of Health and other national-level government agencies (8).

Similar field epidemiology skills are also necessary to enable the animal health workforce to prevent and rapidly detect, respond to and contain outbreaks of endemic and emerging diseases. Several evaluation tools are available to assess these capacities within the veterinary sector, including the Performance of Veterinary Services (PVS) conducted by the World Organization for Animal Health (9), the Joint External Evaluations (JEE) of the World Health Organization, and the Surveillance Evaluation Tool (SET) and Epidemiology Mapping Tool (EMT) of the Food and Agriculture Organization of the United Nations (10–15). Outcomes of these health systems evaluations have often indicated that field epidemiology skills in veterinarians are generally inadequate at both country and regional levels.

To address these gaps, some countries, such as Nigeria, expanded their FETPs to include veterinarians (16). Elsewhere, stand-alone field epidemiology training programs for veterinarians (FETPVs) modeled on those for public health have been developed (2). While some of these are national programs, others are regional and provide training for the workforce of several countries, such as the Regional FETPV in South East Asia (17) and The Regional Field Epidemiology Training Program for Veterinarians (R-FETPV) in the Asia Pacific. These training programs are usually led by non-academic institutions or government ministries, in partnership with international and national organizations and academic institutions (2, 18), and focus on developing the capacities of veterinarians working at the national and sub-national government levels. However, there is not a set of globally agreed upon core competencies that would allow for harmonizing field veterinary epidemiology training curricula across institutes.

A competency is a combination of essential elements of knowledge, skills, and attitudes that are critical to making correct judgements and performing work effectively, and can be accrued through learning, training, and hands-on experience (19, 20). While core competencies have been developed for all the three levels of FETPs, these are lacking for field veterinary epidemiology. Previous competency development processes suggest that a rigorous definition should result from an iterative and participative process involving representatives of a broad spectrum of international experts including field epidemiology practitioners, scholars, and academics (19). Therefore, FAO took the initiative in 2018 to develop with partners a standardized set of core competencies for field veterinary epidemiology for the frontline and intermediate levels. The purpose of this manuscript is to summarize the methodology and outcomes of this process, and to discuss the relevance of the identified competencies and how they can be utilized to develop harmonized curricula for field epidemiology training programmes which can then be adapted to the countries' workforce needs.

## Methodology

The integration of core competencies into the curriculum development process is critical to the success of training programs and serving as the cornerstone for the design and delivery of impactful training programs. The subsequent steps are to assess the current skills and knowledge of the target audience, consisting of independent field officers, researchers, and cadres, through a comprehensive needs assessment.

In 2018, FAO convened two in-person workshops at FAO Headquarters in Rome, Italy. The first three-day workshop was held in February 2018 and was attended by 81 experts. These experts represented FETP and FETPV programs, and other institutions and countries where similar field/applied veterinary epidemiology trainings for veterinarians have been implemented (list available from the corresponding author on request). The specific objectives of this first workshop were to: (a) review existing public health and field veterinary epidemiology programs and share lessons learned, and (b) identify a framework for the development of core competencies that could guide the establishment or strengthening of a standard curriculum for field veterinary epidemiology training. The experts identified the roles and responsibilities of field veterinary epidemiologists based on a set of guiding objectives (Box 1) and proposed a list of competencies for frontline and intermediate levels.

Box 1 **Guiding objectives used during the first workshop organized by the FAO in Rome in 2018 to facilitate the development of the core competency framework and field veterinary epidemiology programs.**

1) Identify the training needs, roles and responsibilities for veterinarians and staff from animal health services in epidemiology, including for those working at district or local levels.
2) Review core competencies in the existing public health applied epidemiology training programs for frontline and intermediate levels.
3) Identify competencies specific for the veterinary workforce, in addition to the existing public health field epidemiology training programs.
4) Discuss the requirements for a standardized core curriculum for FETPV training programs and how it can be harmonized with FETP and other public health field epidemiology programs.
5) Discuss an approach to develop a repository of training modules, presentations, training tools, etc., that can be used by FETPV training programs.
6) Decide the prerequisites for undertaking FETPV training at all three levels and how the prerequisites can be evaluated.
7) Develop guidance to measure progress and assess the skills and knowledge of trainees.

The second three-day workshop was held in July 2018 and was attended by 19 experts (list available from the corresponding author on request). The objective of this second workshop was to develop a set of core competencies for field veterinary epidemiologists for the frontline and intermediate levels, as recommended during the first meeting (21). Core competencies from the CDC (4, 22), TEPHINET (1), and ECDC were used as references (20) to develop a set of core competencies for field veterinary epidemiology training programs. Phrasing of the core competencies was based on the Bloom approach (23) which states that a competency statement should consist of three elements: an action verb, the content, and the context. Specifically, the action verb of the competency describes the observable or measurable performance. The content identifies the subject, type of performance, and the specific task which should be neither too narrow, nor too broad. The context describes the working environment and captures any relevant limitations or specific conditions.

To facilitate engagement among the experts, we encouraged active participation and facilitated discussions. For example, during the first workshop, FETP training programs were reviewed, and lessons learned were discussed with the objective of identifying gaps. The workshops created a collaborative and inclusive environment where all experts could contribute their knowledge and expertise. The use of various participatory techniques helped to ensure that all participants were able to actively participate and engage in the discussions. During the second workshop small group discussions were facilitated to encourage a more in-depth exchange of ideas.

The competencies were further refined and developed after the second workshop. A Technical Consultative Group (TCG), mostly consisting of participants in the second workshop, was set up to monitor this process. Updated versions of the competency document were circulated to the TCG electronically.

This process involved three rounds of feedback and revision to refine and improve the list of core competencies, also we had continuous communication with experts to gather their input and feedback on the list of core competencies during the process. The use of an iterative process helped to ensure that the list of core competencies was comprehensive and reflective of the needs and requirements of field epidemiology training. By incorporating the input and feedback of experts at each stage of the process, we were able to reach a mutually agreed-upon list of core competencies that was representative of the needs of the field.

## Results

The workshops identified the training needs for veterinarians working at the frontline and the intermediate levels, which enabled the development of core competencies for candidates receiving training at these levels. The identified roles and responsibilities grouped into seven areas are listed in Table 1.

**Table 1:** Roles and responsibilities for field epidemiologists at the frontline and intermediate levels.

**Area**	**Frontline workforce**	**Intermediate workforce**
Preparedness	• Apply Standardized Operating Procedures (SOPs) and regulations • Situational awareness • Value chain mapping	• Cross-border trade, threats, relations
Surveillance	• Identify single cases • Identify an outbreak • Disease reporting	• Collate, analyse, and interpret surveillance data • Plan and implement local surveillance • Qualitative risk assessment • Risk-based surveillance
Outbreak investigation	• Participate in an outbreak investigation • Prompt response • Containment, biosecurity	• Outbreak investigation • Response
Epidemiological methods	• Data entry/capture • Data summarisation	• Design and conduct surveys • Descriptive epidemiology • Use data analysis software • Participatory disease search • Mapping • Value chain analysis
Communication	• Communicate effectively with the community • Cross-sectoral coordination • Two-way vertical and horizontal communication and coordination • Communication with wide audience/public	• Prepare surveillance report • Prepare outbreak report • Present report to stakeholders • Risk communication • Active listening • Scientific presentation
Laboratory methods	• Collection and handling of samples • Biosafety and biosecurity	• Design laboratory forms • Complete laboratory forms • Interpret laboratory results
Professionalism	• Ethical behavior • Teamwork	• Ethical behavior • Teamwork

The final proposal comprised a three-tier field veterinary epidemiology training program that can be tailored to the needs and context of countries. Core competencies for the frontline and intermediate levels required for field veterinary epidemiologists working at the frontline and intermediate levels are presented in Boxes 2, 3, and complete information is presented in Supplementary materials S1, S2.

Box 2 **Summary of the 13—field veterinary epidemiology core competencies for the frontline level, grouped into three domains.**

- **Domain 1: Epidemiological surveillance and studies**

1. Describe the purpose of surveillance and characteristics of an effective surveillance system.
2. Identify cases and clusters of diseases/syndromes of animal/public health interest.
3. Monitor and assess the quality of local animal health surveillance data.
4. Summarize, analyse, and interpret surveillance data.
5. Produce surveillance summary reports providing information that is useful for decision-making.

- **Domain 2: Field investigation, preparedness and response**

1. Apply regulations and standard operating procedures in the context of preparedness.
2. Apply proper biosafety and biosecurity methods.
3. Conduct outbreak/health event investigations.
4. Contribute to the diagnosis of cases.
5. Contribute to disease control activities.

- **Domain 3: One Health, communication, ethics and professionalism**

1. Follow the One Health approach.
2. Communicate effectively with technical and non-technical audiences.
3. Act in accordance with the accepted standards of ethics and professionalism.

Box 3 **Summary of the 13—field veterinary epidemiology core competencies for the intermediate level, grouped into three domains.**

- **Domain 1: Epidemiological surveillance and studies**

1. Evaluate an animal or public health surveillance system.
2. Analyse surveillance data using appropriate epidemiological methods.
3. Conduct a prevalence survey or an epidemiological study.
4. Conduct a participatory disease search/investigation in a community.
5. Conduct value chain mapping.
6. Apply basic methods of animal health economics.
7. Participate in a team for conducting risk assessment or demonstrating disease freedom.

- **Domain 2: Field investigation preparedness and response**

1. Plan and conduct an outbreak investigation.

- **High level domain 3: One Health, communication, ethics and professionalism**

1. Be proficient in oral and written communication.
2. Provide adequate mentorship and staff support.
3. Adhere to ethical principles.
4. Apply epidemiological principles to disease prevention and control.
5. Follow the One Health approach.

The proposed frontline level aims to provide sub-district level veterinarians with basic skills in 13 competencies related to routine surveillance, data collection and analysis, disease monitoring, outbreak investigation and response activities (Box 2). It is four-months long, including one month of in-person training and a three-month mentorship period during which they are to complete one or more case studies to acquire hands-on experience of the core competencies.

The proposed intermediate programme is nine months long and targets veterinarians working at a sub-national level (e.g., provincial/state or district staff). This programme aims to provide training on 13 intermediate-level competencies required to take a lead role in outbreak investigations, conducting and assessing surveillance activities, managing and analyzing surveillance data, and using epidemiological methods for disease investigation (Box 3).

Besides developing the competencies, the TCG recommended to develop (a) a repository of training resources in field veterinary epidemiology that can be adapted by any FETPV program to suit their needs; and (b) assessment activities to evaluate the achievement of the proposed competencies.

To effectively integrate these competencies into training curricular, several steps can be followed. First, the core competencies should be translated into specific, measurable, and achievable learning objectives. These objectives can then be used as the foundation for building lessons or training models. A variety of teaching methods, including classroom lectures, problem-based learning, case studies, exercises, and field projects for practical applications, can be incorporated into the curriculum to impart the necessary knowledge, skills, and attitudes. Additionally, the curriculum should be organized to facilitate the systematic development and application of the core competencies at frontline, and intermediate levels. To measure mastery of these competencies, assessments and evaluations aligned with the learning objectives should be developed and integrated into the curriculum. To ensure the mastery of competencies, various assessment methods such as tests, demonstrations, and simulations can be utilized.

## Discussion

Field veterinary capacities in countries and regions are lacking or insufficient (24), with recent estimates indicating that in Africa, there are only 1,900 epidemiologists compared to the 6, 000 needed as per the Global Health Security Agenda (25). Therefore, strengthening capacity and building human resources are essential to enhance disease preparedness and improve chances of successful disease control (26, 27). FETPVs have been implemented in several countries mainly in Asia to achieve this, but core competencies for field epidemiologists had not been identified, leading to the development of different training curricula. This paper documents the first initiative to develop a standardized set of core competencies for field veterinary epidemiology training at both frontline and intermediate levels, based on contributions from over 80 experts representing leading organizations in field epidemiology globally. These core competencies captured the skills, knowledge, and attitudes required for field veterinarians and were grouped into three domains: epidemiological surveillance and data collection, field investigations, and One Health skills and communication.

The first domain focuses on epidemiological surveillance and studies. Surveillance is defined as the ongoing and systematic collection, analysis, interpretation, and timely communication of health-related data to all those who contribute information (28, 29). Frontline core competencies in this domain include the ability to describe the purpose and characteristics of surveillance, monitor data quality, summarize and interpret data, and produce reports. At the intermediate level, these competencies also extend to conducting epidemiological studies, participatory investigations and risk assessments, analyzing data, and understanding animal health economics.

Surveillance is critical for early detection of exotic or re-emerging pathogens, monitoring endemic diseases, and demonstrating freedom from disease (30, 31). Furthermore, information generated by surveillance activities improves our understanding of the economic and social impact of diseases, thus informing disease prioritization and resource allocation exercises (32, 33). Notwithstanding the recognition that epidemiological surveillance constitutes the cornerstone of a robust animal health system, previous assessments have shown that national veterinary services often lack epidemiology workforce capacities to conduct surveillance. In many countries, active surveillance plans are rare, while passive surveillance systems exist and often lack performance indicators or monitoring and evaluation frameworks (10). Furthermore, if frontline health workers do not fully understand the rationale behind data collection, they may be reluctant to participate in surveillance activities (34). Consequently, the development of human resources through training and hands-on experience will contribute toward an enabling environment that facilitates the implementation of surveillance activities (32).

The second high-level domain, which focuses on field investigation, preparedness and response, is the most extensive. This highlights the fact that the field workforce needs skills to respond to a broad range of animal health threats, including zoonotic and transboundary animal diseases, and that field investigations incorporate numerous and varied tasks. Core competencies within this domain include applying regulations and biosecurity principles, conducting outbreak investigations, and contributing to disease diagnosis and control activities. While some of these competencies can be learned through theory, field experience is required and essential to grasp the complexity of other tasks such as defining and identifying cases, collecting and shipping samples to laboratories for testing, and applying biosecurity and biosafety measures.

The emergence of new digital technologies and increased penetration of mobile networks in many previously inaccessible regions can therefore be leveraged by field epidemiologists to facilitate data collection (35). In this regard, FAO has been supporting the surveillance capacities of countries to use the Event Based Mobile Application (FAO EMA-i) and Global Animal Disease Information System (EMPRES-i) for collecting and storing data on disease events (36). It is, therefore, important that field investigation training also includes elements of computer technology and informatics so field epidemiologists can make optimal use of the digital technologies available (1).

One Health, communication, ethics and professionalism constitute the third high-level domain. The OH approach has been gaining momentum over the past two decades, in recognition that 60–70% of emerging infectious diseases, including most recent outbreaks, are zoonotic in nature and require an integrated approach (37). It was therefore considered important to include OH to ensure the development of a field veterinary workforce that advocates for the inclusion of OH approaches to address health threats shared across multiple sectors. More recently, the need for a OH approach to address complex and multi-disciplinary challenges was also advocated for during the 2021 Nobel Prize Summit “Our Planet, Our Future” (38) and endorsed in the G7 Carbis Bay Health Declaration (39).

Other competencies within this OH domain include effective communication, providing mentorship, and following ethical principles. “Soft” organizational capacities, including communication, diplomacy, networking and leadership are important to engage with community members, build trust, and improve health systems (26, 40, 41). Furthermore, lack of communication is often listed as one of the primary challenges to developing integrated systems (42). Equipping the field workforce with such functional skills will allow them to develop the intellectual and social capacity to navigate the various challenges they may encounter during their work. In many countries, other animal health professionals and paraprofessionals also play a critical role in delivering animal health services and maintaining the health and wellbeing of animal populations. The core competencies were specifically designed for veterinarians for frontline and intermediate levels, but they can certainly be adapted for use by other animal health professionals and paraprofessionals (frontline) as well in the curriculum development for the target group. Moreover, academic institutions can consider incorporating these core competencies into the development of undergraduate veterinary curriculum and training programs in the future.

This set of globally agreed-upon core competencies can also be used to develop tools to assess training needs and gaps in field epidemiology training programs within the national veterinary services. Such assessments can then inform the development of the training curricula to ensure that they are tailored to the specific needs and socioeconomic context of the country. Additionally, case studies to be completed during the mentorship process can then be designed to focus on the country's priority diseases or pressing needs. As an example, FAO In-service Applied Epidemiology Training for Veterinarians (ISAVET) have adapted these core competencies to develop a curriculum for frontline field epidemiology training in Africa (43, 44). This training program has now been in place since 2019 and has been developing a cadre of skilled field frontline workforce who are now responding to outbreaks and contributing to disease preparedness, prevention, and control across the African continent. Similarly, the Asia Pacific Consortium of Veterinary Epidemiology (APCOVE) has developed 36 eLearning modules and case studies, loosely based on these core competencies, for intermediate level field epidemiology training programs. These modules have been successfully piloted with more than 100 field epidemiology trainees from seven countries in the Asia Pacific.

Competency sets generally have a lifespan of three to five years, and therefore need to be updated regularly depending on emerging challenges (45). Given recent trends, competencies related to information technology, disease intelligence (46) and antimicrobial resistance surveillance may soon need to be considered. While the core competencies described here have been grouped within three high-level domains, future iterations may consider creating more domains to narrow the focus of each domain. As an example, the first high-level domain could be split into three separate domains for surveillance, epidemiological studies, and data management and analysis, respectively. This is similar to what has been proposed by TEPHINET, where public health surveillance, epidemiological methods, and biostatistics, are listed as distinct domains (1). Similarly, the One Health competencies can be developed into separate domains given their increasing importance (47). Finally, future iterations could also consider distinguishing between competencies that are specific to field veterinary epidemiology, and those that are more generic and that can be applied for training of other close disciplines, as has been proposed by the ECDC (20).

During the expert workshops, ensuring sustainability of such competency-based training programs was identified as a crucial element. Indeed, while certain regional and national FETPV in Thailand, Vietnam and Cambodia have been successful in establishing a long-term program (2), the regional field epidemiology training in South Asia was only implemented for 2 years through a European Union-funded project, and was later discontinued (48). To overcome sustainability challenges, a Training-of-Trainers approach can be adopted, whereby participants go on to become mentors for participants in subsequent training programs. This can also help to instill national ownership of the program while contributing to the development of a national network of field epidemiologists. Alternatively, the APCOVE model involving regional eLearning training combined with in-country mentored hands-on projects could be implemented. This approach is cost-effective and result in strengthening epidemiology capacity in the entire region at a fraction of the cost. Formal recognition and accreditation of the training programs by the national Veterinary Council or academic institutions will also allow for the training to become sustainable (49). Finally, the concurrent development of a workforce capacity development plan will allow to identify needs and gaps for sustainability, and to formulate recommendations accordingly (50).

## Conclusions

This paper describes the iterative process used to develop a set of competencies considered essential for field veterinarians. These core competencies for field veterinary epidemiology can be used to develop harmonized training programs to build and develop the field epidemiology workforce capacity as demonstrated with the establishment of ISAVET and APCOVE programs, for frontline and intermediate tiers, respectively, based on the identified competencies. A limitation of this work is that during this concerted effort, core competencies were developed only for veterinarians at the frontline and intermediate levels. Further work needs to be done to develop competencies for the advanced level, assessment activities to evaluate the achievement of the competencies and a mechanism to ensure quality assurance and conduct accreditation of field epidemiology training programs for veterinarians. Development of mentorship and sustainability guidelines would be helpful in strengthening field epidemiology training to ensure that the graduates are able to conduct outbreak investigation and surveillance activities in their countries to support early detection, quick response and effective containment of disease threats.

## Data availability statement

The original contributions presented in the study are included in the article/Supplementary material, further inquiries can be directed to the corresponding authors.

## Author contributions

JP technical coordination of the workshops, contributed to the development of core competencies, conceptualized, wrote the initial draft, revised and edited the manuscript, coordinated the manuscript writing between co-authors, and coordinated manuscript submission. RD and CR-G contributed to the conceptualization of the manuscript, contributed to the development of core competencies, literature review, data analysis, wrote the initial draft, and revised and edited the manuscript. ND contributed to the conceptualization of the manuscript, contributed to the development of core competencies, wrote parts of revised, and edited the manuscript. FL conceptualization of the manuscript, wrote parts of revised, and edited the manuscript. HA, RA, DB-A, KC, JGu, AH, FL, LL, PM, HR, SSa, CS, IR, CVS, and KW contributed to the development of core competencies. CL, SO, YK, JGi, and BS contributed to review and editing the manuscript. MD coordination of the workshops and contributed to the development of core competencies. SSh and JGu coordination of the workshops, contributed to the development of core competencies, and revised and edited the manuscript. DC contributed to the development of core competencies and discussion and interpretation of results. KS overall supervision, contributed to the development of core competencies, and revised and edited the manuscript. TT overall supervision of the manuscript. All authors contributed to the article and approved the submitted version.
