# Supplementary material for: Development of core competencies for field veterinary epidemiology training programs
Source: Front Vet Sci. 2023 Apr 6;10:1143375. doi: 10.3389/fvets.2023.1143375 (PMC10118009; doi:10.3389/fvets.2023.1143375)
Supplement: Supplementary file 1 [file Data_Sheet_1.pdf]

Supplementary Material

Development of core competencies for field veterinary epidemiology training programs

Julio Pinto[1,2*], Ravi Bandara Dissanayake[1*], Navneet Dhand[1,3] , Cristina Rojo-Gimeno[1†], Laura Cristina Falzon[1], Holy Akwar[1], Rianatou Bada Alambeji[4] , Daniel Beltran-Alcrudo[5], David Mario Castellan[6] , Karoon Chanachai[7], Javier Guitian[8], Angela Hilmers[9], Fairouz Larfaoui[1], Leo Loth[10], Paolo Motta[1], Harentsoaniaina Rasamoelina[11], Stephanie Salyer[12], Sean Shadomy[1,12], Cécile Squarzoni[13], Innocent Rwego[14], Carmen Varela Santos[15], Kachen Wongsathapornchai[16], Caryl Lockhart[1], Sam Okuthe[17], Yaghouba Kane[18], Jeffrey Gilbert[1], Baba Soumare[1], Madhur Dhingra[1], Keith Sumption[1] and Thanawat Tiensin[1]

[1]Food and Agriculture Organization of the United Nations, Rome, Italy, [2]Food and Agriculture Organization of the United Nations, Liaison Office for the United Nations, Geneva, Switzerland, [3]Sydney School of Veterinary Science, The University of Sydney, Camden, NSW, Australia, [4]Ecole Inter-Etats des Sciences et Medecine Vétérinaires, Dakar, Senegal, [5]Food and Agriculture Organization Regional Office for Europe and Central Asia, Budapest, Hungary, [6]Institute for Infectious Animal Diseases, Texas A&M University, College Station, TX, United States, [7]Department of Disease Control/Department of Livestock Development, Ministry of Agriculture and Cooperatives, Bangkok, Thailand, [8]The Royal Veterinary College, Hatfield, United Kingdom, [9]Training Programs in Epidemiology and Public Health Interventions Network, Atlanta, GA, United States, [10]Food and Agriculture Organization Emergency Center for Transboundary Animal Diseases, Hanoi, Vietnam, [11]Epidemic Surveillance and Alert Management, Indian Ocean Commission, One Health Network, Mauritius, [12]Centers for Disease Control and Prevention, Atlanta, GA, United States, [13]Centre de Coopération Internationale en Recherche Agronomique pour le Développement, La Réunion, France, [14]University of Minnesota/One Health Central and Eastern Africa, Makerere University, Kampala, Uganda, [15]Public Health Capacity and Communication Unit, European Centre for Disease Prevention and Control (ECDC), Stockholm, Sweden, [16]Food and Agriculture Organization, Emergency Center for Transboundary Animal Diseases, Bangkok, Thailand, [17]Food and Agriculture Organization of the United Nations, Nairobi, Kenya, [18]Food and Agriculture Organization of the United Nations Regional Office for Africa, Accra, Ghana.

[†]PRESENT ADDRESS: Cristina Rojo-Gimeno, Biological Hazards and Animal Health and Welfare Unit, European Food Safety Authority, Parma, Italy

CORRESPONDENCE: Julio Pinto, Julio.Pinto@fao.org

Ravi Bandara Dissanayake, Ravi.Dissanayake@fao.org

DOI 10.3389/fvets.2023.1143375

Supplementary Material - S1

FRONTLINE CORE COMPETENCIES FOR FIELD VETERINARY EPIDEMIOLOGY TRAINING PROGRAMS

Domain 1: Epidemiological surveillance and studies

1. Describe the purpose of surveillance and characteristics of an effective surveillance system.

a) Describe the role of surveillance in public and animal health practice and for society.

b) Describe the key characteristics and functions of the surveillance system.

c) Describe the role and responsibility of the surveillance system.

2. Identify cases and clusters of diseases/syndromes of animal/public health interest.

a) Recognize cases and suspected clusters of disease or syndromes of animal and public health interest in the field by applying the case definition.

b) Ensure that cases and suspected clusters of disease or syndromes of animal and public health interest are reported to the appropriate governmental authorities.

3. Monitor and assess the quality of local animal health surveillance data.

a) Monitor the timeliness and completeness of data reported from different sources.

b) Check the quality of surveillance data.

c) Provide feedback to improve timeliness, completeness and quality of surveillance data.

4. Summarize, analyse and interpret surveillance data.

a) Describe the rationale for regular review and analysis of surveillance data.

b) Summarize surveillance data using descriptive epidemiological and statistical methods.

c) Present surveillance results using tables, graphs and maps as appropriate.

d) Map the links between value chain actors and product flow in a livestock-associated value chain.

e) Identify and describe trends, patterns, deviations and outliers.

f) List the possible causes of a sudden increase in cases or health or production events.

g) Notify authorities of suspected high-priority diseases and incidence that exceed thresholds.

5. Produce surveillance summary reports providing information that is useful for decision-making.

a) Produce a surveillance summary report including descriptive epidemiology, descriptive statistics, and brief descriptive and interpretative text for key reportable conditions, with recommendations for action as appropriate.

b) Design a surveillance summary report template that can be used periodically.

Domain 2: Field investigation, preparedness and response

6. ***Apply regulations and standard operating procedures (SOPs) in the context of preparedness.***

a) Explain regulations and SOPs relevant to their office and position.

b) Practice situational awareness by monitoring the local environment and conditions.

c) Take actions consistent with regulations and SOPs, recognizing the local environment.

7. ***Apply proper biosafety and biosecurity methods.***

a) Use appropriate personal protective equipment (PPE) when conducting field investigations and handling infectious material.

b) Dispose of infectious material using accepted standards for safety and effectiveness.

c) Use appropriate biosecurity methods and situational awareness when conducting field investigations.

8. ***Conduct outbreak/health event investigations.***

a) Recognize any suspected animal cases of animal and public health interest in their area.

b) Collect individual case/farm information.

c) Actively search for cases in your area based on an established case definition.

d) Conduct follow-up action to the identified cases including trace-forward and trace-backward searches.

e) Participate as a team member in the investigation and verification of a reported or suspected outbreak.

f) Create a line list.

g) Validate data (ensure data quality) including by triangulation.

h) Provide basic descriptive information on cases.

i) Recommend and implement basic outbreak prevention and control measures and make recommendations.

9. ***Contribute to the diagnosis of cases.***

a) Use appropriate field diagnostic methods for case detection and diagnosis.

b) Establish communication with a laboratory to ensure appropriate samples are collected and managed using best practices.

c) Manage specimens – collect, label, package and transport samples for diagnosis using accepted methods, use them and provide input on sample submission forms.

10. Contribute to disease control activities.

a) Apply the principles for disease control and prevention in familiar settings.

b) Describe existing control programmes in your country.

c) Describe role and responsibility in disease control and prevention. Domain 3: One Health, communication, ethics and professionalism

Domain 3: One Health, communication, ethics and professionalism

11. Follow the One Health approach.

a) Identify the sectors that may be involved in addressing a multifaceted health event.

b) Include animal, human and environmental sectors in surveillance, outbreak investigation, preparedness and response to multifaceted health events.

12. Communicate effectively with technical and non-technical audiences.

a) Promote good rapport and open lines of communication with stakeholders including government officials and staff, the public health sector, animal owners and others along the value chain, and the general public.

b) Deliver risk communication messages to diverse audiences.

c) Prepare and deliver a written report or oral presentation to a technical audience.

d) Demonstrate cultural awareness in communication.

13. Act in accordance with the accepted standards of ethics and professionalism.

a) Act in accordance with ethical principles and promote animal and human welfare.

b) Promote inclusivity, diversity and respect for cultural differences.

c) Adhere to policies, processes and procedures for ethical guidelines and principles regarding data collection, use, management and disposal.

d) Manage conflicts of interest in accordance with ethics, principles and guidelines.

e) Interact with colleagues, stakeholders, the general public, and government officials with respect and professionalism.

f) Be an effective team member, adopting the role needed to contribute constructively to the group's accomplishment of tasks.

Supplementary Material - S2

INTERMEDIATE CORE COMPETENCIES FOR FIELD VETERINARY EPIDEMIOLOGY TRAINING PROGRAMS

Domain 1: Epidemiological surveillance and studies

1. ***Evaluate an animal or public health surveillance system.***
   a) Describe the purposes and operation of a surveillance system.
   b) Evaluate the operation and disease-reporting components of a surveillance system.
   c) Identify the strengths, limitations and gaps of a surveillance system.
   d) Make recommendations to improve the performance of a surveillance system.

2. ***Analyse surveillance data using appropriate epidemiological methods.***
   a) Describe surveillance data:
      i) Check surveillance data for data entry errors, typos and implausibility, and validate data.
      ii) Conduct descriptive analyses using surveillance data.
      iii) Create appropriate charts, maps and graphs (epidemic curves) to display data.
   b) Compare measures of disease frequencies using appropriate statistical methods:
      i) Calculate the measures of disease frequency and association.
      ii) Create contingency tables to compare frequencies between subpopulations and measure associations.
      iii) Compare disease frequencies between subpopulations and identify risk factors.
   c) Interpret the results and prepare reports:
      i) Interpret analysis results to identify trends, patterns and risk factors in surveillance data.
      ii) Design and write appropriate audience-specific reports for farmers, animal health authorities and other stakeholders on key findings.
      iii) Present results to colleagues and other stakeholders using appropriate presentation software programs.

3. ***Conduct a prevalence survey or an epidemiological study.***
   a) Design a prevalence survey or an epidemiological study:
      i) Explain the differences between cohort, cross-sectional and case-control study designs and select an appropriate design for a given objective.
      ii) Conduct a literature review to support the development of study hypotheses.
      iii) Define the study objectives.
      iv) Specify a case definition or selection criteria.
      v) Specify the target and the study populations as well as the units of interest.
      vi) Calculate sample size and plan a sampling strategy.
      vii) Identify and prepare data-collection tools, including sample submission forms, questionnaires, and data entry spreadsheets or a database.
      viii) Identify key biases that might affect study findings and consider approaches in study design to minimize these biases.

ix) Obtain approval from an animal and/or a human ethics committee, as appropriate.

b) Conduct the study:
   i) Establish and manage a field team.
   ii) Train staff to apply and use appropriate biosafety and biosecurity measures.
   iii) Select villages/farms/animals or other units of interest from the sampling frame using the planned sampling strategy.
   iv) Collect samples and data by using appropriate data-collection methods.

c) Analyse and interpret data and prepare a report:
   i) Enter data into a spreadsheet or database and check for data entry errors.
   ii) Conduct appropriate statistical analyses depending on the type of study and the study objectives and interpret the results.
   iii) Make evidence-based conclusions and recommendations within reason.
   iv) Prepare an audience-appropriate report for animal health authorities and present the findings to peers and other stakeholders.
   v) Prepare an abstract/manuscript for submission to a workshop or conference, or publication in a scientific journal.

4. ***Conduct a participatory disease search/investigation in a community.***

a) Prepare for a participatory disease search/investigation:
   i) Establish a team.
   ii) Collect background data about the communities from secondary sources. iii) Organize a meeting with the members of the target community.
   iv) Prepare all questionnaire material and equipment including a checklist for the topics to be discussed in the interview.

b) Conduct participatory disease appraisal:
   i) Conduct semi-structured interviews with participants.
   ii) Obtain animal-production and health information including animal ownership, species and husbandry system using proportional piling techniques and triangulation.
   iii) Collect information about current and historical disease problems.
   iv) Conduct triangulation to validate data and disease reports and link data to routine surveillance data.

c) Analyse and interpret data with planned follow-up:
   i) Analyse data and describe temporal trends and seasonal/spatial patterns.
   ii) Confirm the occurrence of any active disease by clinical and/or laboratory investigations.
   iii) Make recommendations for further investigations or for disease prevention and control.

5. ***Conduct value chain mapping.***

a) Preparation:
   i) Specify the objectives of value-chain mapping. ii) Establish a team to conduct value-chain mapping.
   iii) Conduct key informant interviews to identify key actors in the chain.

b) Conduct interviews and analyses:
   i) Conduct in-depth semi-structured interviews to develop the value-chain map.

ii) Identify actors, activities, seasonality, volumes, product flows and links between them.
iii) Identify gaps in data and information in the value chain exercise. iv) Identify disease transmission risk points/processes in the value chain.
v) Prepare a value-chain analysis/mapping report with recommendations for disease prevention and control.

6. ***Apply basic methods of animal health economics.***
  a) Describe the application of economics for animal production and health.
  b) Prepare a partial farm budget in relation to a change in a farming practice.
  c) Calculate gross margins for a farming enterprise.
  d) Conduct a cost-benefit analysis of an animal disease control programme.

7. ***Participate in a team for conducting risk assessment or demonstrating disease freedom.***
  a) Describe the framework and the basic steps for conducting a risk assessment:
    i) Discuss the objective(s) of risk assessment.
    ii) Describe how to identify hazards.
    iii) Describe the risk pathways by which the undesirable event could occur. iv) Identify gaps in data and knowledge.
    v) Discuss the approach for qualitative risk assessment.
    vi) Prepare a risk assessment report with management approaches for the identified risk.
  b) Explain the approach for demonstrating disease freedom:
    i) Define the objective(s) of demonstrating disease freedom.
    ii) Define the population.
    iii) Calculate sample sizes required for achieving a given confidence.
    iv) Select sampling units (villages/farms/animals) depending on the study design.
    v) v) Collect and submit samples. vi) Analyse data and interpret the results.

Domain 2: Field investigation preparedness and response

8. Plan and conduct an outbreak investigation.
  a) Prepare for an outbreak investigation:
    i) Apply SOPs in an outbreak investigation.
    ii) Prepare logistics for sample collection (transportation, culture media, submission kits, etc.).
    iii) Establish and coordinate an outbreak investigation team and arrange PPE for all team members.
    iv) Train team members in sample collection and in the use of PPE.
    v) Create generic data-collection tools such as sample submission forms and outbreak investigation questionnaires.
  b) Conduct field investigation: find cases, and collect samples and data:
    i) Confirm the existence of an outbreak.
    ii) Develop and apply case definitions based on clinical signs and/or diagnostic test results as appropriate to classify animal units into cases and non-cases.
    iii) Find cases and collect data about the numbers, risk factors, types and locations of cases and non-cases and the dates of occurrence of cases.

- iv) Collect samples from both cases and non-cases as appropriate and submit them to the laboratory for diagnostic purposes.
- v) Conduct trace-back and trace-forward operations, if necessary.
- vi) Determine the source/cause of the outbreak to inform preventative actions and measures as needed.

c) Analyse and interpret data using epidemiological approaches:

- i) Enter and validate data collected from the field investigation into a spreadsheet or a database programme.
- ii) Interpret the diagnostic test results for samples submitted to the laboratory.
- iii) Prepare and interpret an epidemic curve to describe the outbreak. iv) Describe animal disease spatial patterns using appropriate techniques.
- v) Analyse data, cross-tabulate data, and calculate and compare attack risk between different subgroups/populations.
- vi) Develop and test hypotheses for the potential causes and/or source of the outbreak based on field investigation, laboratory results (if available) and data analysis.
- vii) Develop and apply preliminary control/prevention strategies to contain the outbreak.

d) Report and follow-up:

- i) Develop evidence-based recommendations and an implementation plan for disease prevention and control measures.
- ii) Prepare and submit a preliminary report to stakeholders, including farmers and veterinary authorities, with recommendations to control the outbreak.
- iii) Present the findings at conferences and official or professional/industry meetings.

Domain 3: One Health, communication, ethics and professionalism

9. Be proficient in oral and written communication.

a) Prepare departmental and extension educational reports and materials:

- i) Develop educational materials for producers and market chain actors.
- ii) Prepare animal health guidance reports in plain language for farmers based on outbreak investigation, surveillance or other epidemiological activities.
- iii) Prepare reports for animal health authorities or peers based on surveillance, data analysis and other epidemiological investigations.
- iv) Apply the basic principles of risk communication when communicating the message to farmers and other stakeholders.
- v) Use modern communication tools including email, tele- and video-conferencing tools.

b) Present at a meeting, workshop or conference:

- i) Write an abstract/manuscript for submission to a conference or publication in a scientific journal.
- ii) Prepare an oral presentation using presentation software.
- iii) Present the oral presentation to the scientific audience.

10. Provide adequate mentorship and staff support.

a) Mentor and train frontline or junior staff:

i) Provide technical support and advice to FETV trainees or junior staff in the field or in-service projects.
ii) Conduct technical reviews of their work and provide advice. iii) Provide constructive feedback to supervised staff and team members.

11. ***Adhere to ethical principles.***
a) Act in accordance with accepted standards of ethics and professionalism:
i) Respect and appreciate colleagues and stakeholders (e.g. farmers) including diversity (gender, ethnicity, professional background).
ii) Build trust and integrity in all professional transactions with partners/stakeholders.
iii) Perform the assigned task and procedure effectively and in a timely manner. iv) Initiate and participate in team-building exercises. v) Provide feedback as needed.
vi) Adhere to ethical principles and guidelines regarding human and animal welfare; data collection, use, protection and confidentiality; and conflicts of interest.

12. ***Apply epidemiological principles to disease prevention and control.***
a) Apply epidemiological principles for disease transmission and risk factors to the development of disease prevention and control programmes.
b) Use biosecurity principles to prevent and control diseases at the farm, regional and national levels.

13. ***Follow the One Health approach.***
a) Communicate with a range of stakeholders.
b) Follow the One Health approach in developing a disease control programme for a zoonotic disease.
c) Include animal, human and environmental sectors in dealing with a zoonotic disease event.
d) Participate in a team of professionals from a range of disciplines.
e) Lead a team involving professionals from animal, human and environmental sectors.
